# Harnessing the Power of Precision Medicine and Novel Biomarkers to Treat Crohn’s Disease

**DOI:** 10.3390/jcm12072696

**Published:** 2023-04-04

**Authors:** Ofra Aviva Kriger-Sharabi, Uri Kopylov

**Affiliations:** 1 Department of Gatsroenterology, Assuta Ashdod Medical Center, Affiliated to The Ben-Gurion University (BGU) Medical School, Ashdod 7747629, Israel; 2 Department of Gastroenterology, Sheba Medical Center, Tel Hashomer, Affliated to Sackler School of Medicine, Tel Aviv University, Tel Aviv 6997801, Israel

**Keywords:** Crohn’s disease, Inflammatory Bowel Diseases, biomarkers, precision medicine, patient stratification, microbiome, GWAS, multi-omics, therapeutic goals

## Abstract

Crohn’s disease (CD) is a chronic inflammatory condition that affects the gastrointestinal tract. It is part of a spectrum of inflammatory Bowel Diseases (IBD). The disease is complex, characterized by significant inter and intra-individual heterogeneity, which contributes to a diverse and multifaceted portrayal of the disease. Consequently, applying specific and accurate treatment is challenging, and therapeutic success rates remain disappointing and insufficient. In recent years, significant advances in the therapeutic potential of CD have been made. Hope has been provided by these developments in the form of an expanding treatment toolkit. However, even with these beneficial adjustments, patients are frequently treated using an ineffective “one size fits all” treatment protocol, ultimately leading to a plateau in drug effectiveness and a decline in overall treatment success rates. Furthermore, with the advancement in the genome-wide association study, in combination with significant bioinformatic developments, the world of medicine has moved in the direction of personalized, tailored-treatment medicine, and this trend has not escaped the world of IBDs. Prediction models, novel biomarkers, and complex algorithms are emerging and inspiring optimism that CD patients will be treated with “precision medicine” in the near future, meaning that their treatments will be selected based on the patient’s various unique features. In this review, we will outline the current diagnostic and therapeutic limitations that lead to a glass ceiling effect and thus send us in pursuit of discovering novel biomarkers. We will illustrate the challenges and difficulties in discovering relevant and innovative biomarkers and implementing them into everyday clinical practice. We will also heighten the progress made in practicing personalized medicine for CD patients and shed light on future directions and horizons.

## 1. Introduction

Inflammatory Bowel Diseases (IBD) are chronic inflammatory conditions of the gastrointestinal tract that encompass two main clinical entities: Crohn’s disease and ulcerative colitis. Although Crohn’s disease (CD) and ulcerative colitis (UC) have historically been studied together because they share common features (mutual symptoms, equivalent structural bowel damage, and shared therapies), it is now clear that they represent two distinct pathophysiological entities [1]. Inflammatory Bowel Diseases (IBDs) are relapsing and remitting diseases with varying disease trajectories and consequences. In recent years, the prevalence of these diseases has been steadily rising. Frequently, these disorders have lead to long-term morbidity, with many downstream sequelae [2]. However, not all patients suffer from an aggressive disease phenotype. There are patients whose disease follows a relatively indolent path that requires limited intervention and results in few significant complications, including anemia, chronic abdominal pain, and a reduced quality of life. Others may suffer from a devastating, perforating disease that necessitates intensive biologic therapy and surgery [3]. Distinguishing between these distinct phenotypes is crucial in applying adequate therapy to these seemingly different patient populations and underscores the need for proper disease classification.

CD, (much like ulcerative colitis) is a heterogeneous disease, with many clinical, biochemical, endoscopic, and histologic variations, thus rendering it a multi-faceted disease [4]. This variability makes it difficult to predict a patient’s clinical trajectory, prognosis, and response to treatment. Accordingly, we can no longer approach all CD patients equally and the days of “one size fit all” treatment are nearing an end [5,6]. Furthermore, the incidence and prevalence of IBD are increasing worldwide, particularly in association with rapid and drastic changes in industrialization and social behavior in newly westernized countries [7]. Studies evaluating the burden of CD highlight the negative impact of active CD on the patient’s quality of life, especially regarding daily activities and overall well-being [8]. At the turn of the 21st century, IBD has become a global disease. This rise in IBD prevalence and diversity enhances the need to achieve deeper levels of disease control, innovate disease treatment pathways, and provide more “precision” in our personalized treatments.

## 2. Current Diagnostic Approaches and Limitations

Due to the diversity of CD, which may present in an array of clinical, endoscopic, and histologic features, diagnosing the disease correctly and promptly can be challenging. Many patients’ diagnosis is delayed for years, and this can lead to irreversible bowel damage and reduced quality of life. Historically, the mainstay of gastroenterologists’ clinical management relied on clinical parameters that had low accuracy and little predictive value. Furthermore, many of the current serologic markers used for diagnosis are imprecise and have low sensitivity and specificity. Diagnosis using invasive procedures (e.g., colonoscopy) is expensive, time-consuming, and less desirable for patients for obvious reasons. Thus, the need for innovative diagnostic approaches has become crucial. These factors have led gastroenterologists to avoid using clinical predictors and to use validated biomarkers to identify and forecast illness outcomes and help customize treatment algorithms [9].

In order to assess potential novel diagnostic pathways and biomarkers, we must reflect on the extreme complexity of the pathogenesis that leads to the development of IBD, since many of these can be considered targets for diagnosis, prediction, and therapeutic opportunities.

## 3. IBD Has a Complex and Multifactorial Pathogenesis Which Leads to Heterogeneous Disease Phenotypes

Although it is becoming increasingly clear that the pathophysiological processes that cause such a wide range of illness phenotypes must be extremely complicated and intertwined, the precise pathogenesis of IBD remains a mystery. Both CD and UC are associated with a variety of pathogenic variables, such as environmental changes, a variety of susceptibility gene variations, an aberrant gut microbiota in both quality and quantity and a dysregulated immune response [10,11]. The disease complexity is the product of a vast array of various, interacting variables. Still, the exact events initiating and triggering inflammation have not been deciphered.

Furthermore, it is still unclear precisely how these circumstances result in the diverse disease manifestations observed in IBD patients.

### 3.1. Genomic and Epigenomic Alterations

Over 200 IBD-associated susceptible genes have been identified via large-scale genome-wide association studies (GWAS) and transethnic association studies [12]. Many loci are shared by UC and CD and may also be found in other immune-mediated inflammatory disorders [13]. However, IBD development cannot be fully explained by genetics alone, as only 20–25% of all IBD cases can be accounted for by these polymorphisms [14]. Epigenetics, a newly emerging field that serves as a bridge connecting genetics and the environment, also sheds a bright light on IBD pathogenesis [15].

The mechanisms through which the affected IBD-susceptible genes contribute to the disease development include several critical pathways involving T cell signaling, innate immunity, lymphocyte regulation, cytokine release, and influence on intestinal barrier defense robustness [10,16]. IBD ultimately develops as a result of a combination of these genetic defects and environmental factors.

### 3.2. IBD Interactome and Exposome

IBD is viewed as an archetypal complicated disease. This notion is attributed to the vast heterogeneity of clinical manifestations that stem from extremely complicated pathogenic mechanisms [14]. Environmental influences (termed ‘exposome’), such as early life experiences, environmental factors, and food, have all been linked to the emergence of IBD [11]. An initiative in the form of defining an “IBD interactome” proposes the idea of a global disease network, in which specific pathogenic elements (‘-omes’) are all integrated, in order to further define the distinct and individual complex pathogenesis of the disease and assist in the diagnosis and treatment of IBD [14].

### 3.3. The IBD Gut Microbiome

Fascinatingly, gut microbiota, a field that has continuously sparked numerous researchers’ enthusiasm over the past decade, is now recognized to be one of the most significant environmental factors in IBD pathogenesis [17]. The human gut microbiota comprises 10–100 trillion microorganisms, including bacteria, viruses, protozoa, and fungi, among which bacteria are the most abundant, with a density of 1011–1012 cells/mL [18]. Many elements of host homeostasis, including nutrition, immunology, metabolism, and defense mechanisms, are fundamentally influenced by the more than 1000 species of bacteria that inhabit the gastrointestinal system. It is well known that the composition and diversity of the gut microbiota are crucial to the human overall health state and to maintaining a physiological hemostasis status quo. It has been proposed that a decline in microbiota diversity, which causes an imbalance between beneficial and harmful bacteria taxa, damages the gut microbiome, and increases the susceptibility of the microenvironment to the development of IBD [18,19]. This is so much so that microbiome diversity is considered a ‘sine quo non’ of general well-being [18,19].

#### 3.3.1. The Chronic Dysbiosis Theory

There is a bi-directional communication between the gut barrier protection mechanisms and the gut microbiota. The impaired barrier function of the gut epithelium, coupled with reduced mucin expression and lessened anti-microbial activity, results in a thinner barrier between the epithelium and gut microbiome; this enables a more hostile microbiota population [19,20]. Consequently, a drop in the microbiota diversity, which results in the imbalance between beneficial and harmful bacteria taxa, damages the intestinal microbiome, leading to a more susceptible microenvironment; this has been implicated in the development of IBD. This condition has been termed “leaky gut” (a dysfunctional mucosal barrier) and is the hallmark of several GI barrier-related diseases [21]. In the most thorough study of the microbiome in IBD to date, the longitudinal profiles of 132 patients were made, which showed alterations in microbial transcription and metabolite pools, as well as increases in the temporal variability linked to disease activity [22]. Ultimately, the altered gut microbiota results in a state of ‘chronic dysbiosis’ which, in turn, can lead to many gastrointestinal and non-gastrointestinal disorders, including IBD [23]. The predilection of inflammation for anatomical regions with relative fecal stasis (terminal ileum and rectum) and the efficacy of fecal diversion as a treatment for Crohn’s disease are additional pieces of evidence that confirm the role of the gut microbiota in IBD etiology [24].

A number of treatment strategies using dietary or microbial therapies have been developed in order to treat dysbiosis, as a result of the idea that inflammation in IBD may be triggered by the gut microbiota. Probiotics, antibiotics, specified enteral nutritional therapy (ENT), and fecal microbial transplantation are a few examples (FMT). FMT is regarded as an unpolished type of bacteriotherapy that makes use of a healthy donor’s varied gut bacteria flora. FMT is seen as a possible treatment for diseases connected to the microbiota. FMT has attracted significant interest for use in IBD [25] because of its effectiveness in the treatment of refractory Clostridium difficile infection. FMT has been demonstrated to be more successful than the placebo in inducing clinical and endoscopic remission in adults with ulcerative colitis in several randomized, placebo-controlled studies [26,27]. The exact patient cohort that will benefit from this intervention has not yet been identified, and there are still conflicting data regarding the efficacy and safety of FMT in IBD patients.

#### 3.3.2. Unique Characteristics of the Gut Microbiota in IBD Patients Can Predict Disease Phenotypes and Responses to Treatment

The typical gut microbiota seen in IBD patients is characterized by a decrease in species richness in many of the commensal and beneficial fecal bacteria, such as Firmicutes and Bacteroidetes, and by an increase in or bloom of harmful Proteobacteria [17]. As it has been shown that the baseline microbiota differs between patients who respond to treatment and those who do not, thus a specific microbiota signature may be viewed as a biomarker of disease phenotype and may also be helpful in determining a patient’s response to therapy [28]. Another study also suggests that a lower abundance of diverse microbial families in IBD patients could predict stricturing and penetrating disease phenotypes [29]. There is still an ongoing debate about whether chronic dysbiosis/reduced diversity is a cause or a consequence of GI disorders. This is yet to be determined [28]. 

The unique and diverse characteristics of the gut microbiota in IBD patients are summarized in Table 1. 

## 4. Exploring New Frontiers in IBD Management

### 4.1. The Rise of Personalized Medicine: A New Era of Healthcare

The realization that the future of IBD treatment must incorporate a personalized approach to disease management, denoting that ‘the right medicine may be administered to the right patient at the right time’, has increased recently. This is in parallel with other health fields [30].

This personalized approach centers on the exact understanding of the underlying molecular processes driving the disease, combined with the recognition that patients have unique disease features that must be taken into consideration. Personalized therapy has been established in oncology in recent years, utilizing unique biomarkers that can reliably predict prognosis and response to therapy. This breakthrough has sparked a desire for implementing personalized medicine across many disease disciplines, including IBD [31]. Yet, applying this strategy in other medical specialties (beyond the oncology field) has been slower than anticipated. This is largely attributed to the difficulty of identifying specific biomarkers in complex, multi-faceted diseases.

#### Personalized vs. Precision Medicine

It is important to note that while the concepts of personalized medicine and precision medicine are very similar, they are not synonyms. Personalized treatment medicine depends on factors such as the patient’s age, gender, decision-making process, availability of resources, and level of mobility, as well as the presence of comorbidities. It focuses on adjusting treatment to individual differences and is more in line with traditional physician values. such as developing rapport, gaining trust, and applying clinical skills. In an attempt to support better decision-making processes by having in-depth knowledge of the molecular causes of a patient’s disease, precision medicine utilizes a multidisciplinary data-driven approach [32]. Therefore, the opportunity to advance personalized precision medicine in IBD has been dramatically improved by the recent developments in large-scale biological databases. These include the human genome sequence, the new world of “omics” (proteomics, metabolomics, genomics, diverse cellular assays, etc.), mobile health technology, and computational tools for analyzing large sets of data [33].

### 4.2. The Era of Disease-Modifying Endpoints

Although it was once considered the primary goal of IBD treatment, symptom control is now considered an unsatisfactory outcome. As knowledge and understanding of the complexity and long-term sequela of Crohn’s disease expanded, it became clear that disease management had to shift gears and not focus solely on short-term endpoints. In order to optimize disease treatment and surveillance, and prevent long-term disability, the 2015 Choosing Therapeutic Targets in Inflammatory Bowels Disease (STRIDE) consensus and its 2020 amendment (STRIDE 2) proposed a management algorithm for Crohn’s disease (CD) and ulcerative colitis (UC) [34,35]. With this “treat to target” approach, predetermined treatment goals are defined, regular monitoring is carried out accordingly, and if predefined goals are not achieved, the therapy may be changed or optimized. The ultimate goal is to change the course of the disease. Treatments or interventions that modify the disease’s underlying pathophysiology and have a favorable impact on the disease’s long-term course are referred to as “disease modification” [36]. This is in parallel with the evolving management strategies of other progressive, chronic diseases that are all moving in the same direction, determined to not settle for insufficient clinical outcomes [37].

#### The Search for Novel Biomarkers

A biomarker is defined as a substance that offers a quantifiable signal of the presence and/or severity of a disease or an organism’s physiological state. A biomarker’s holy grail must include disease specificity, a strong correlation with disease severity, and the ability to offer both prognostic and diagnostic data [9]. Moreover, a useful biomarker should also be affordable, simple to use, sensitive, and non-invasive. There is presently no single “gold standard” biomarker for diagnosing IBD, assessing the extent of the disease burden, or evaluating a patient’s response to treatment. Physicians still rely on a combination of clinical symptoms, laboratory indices, radiological imaging, endoscopy, and histological biopsies of tissue specimens to assess disease activity and make treatment decisions accordingly [9,38]. However, despite recent advancements in the form of the newly identified biomarkers, integrating these biomarkers into everyday clinical practice is time-consuming and difficult, as shown in other conditions. This is primarily due to the fact that our understanding of the complex molecular pathways that underlie disease heterogeneity is currently lacking.

It is now widely recognized that identifying patient-specific genetic and molecular signals is one of the cornerstones for developing high-quality biomarkers, which can be integrated into everyday clinical practice.

**Figure 1:**
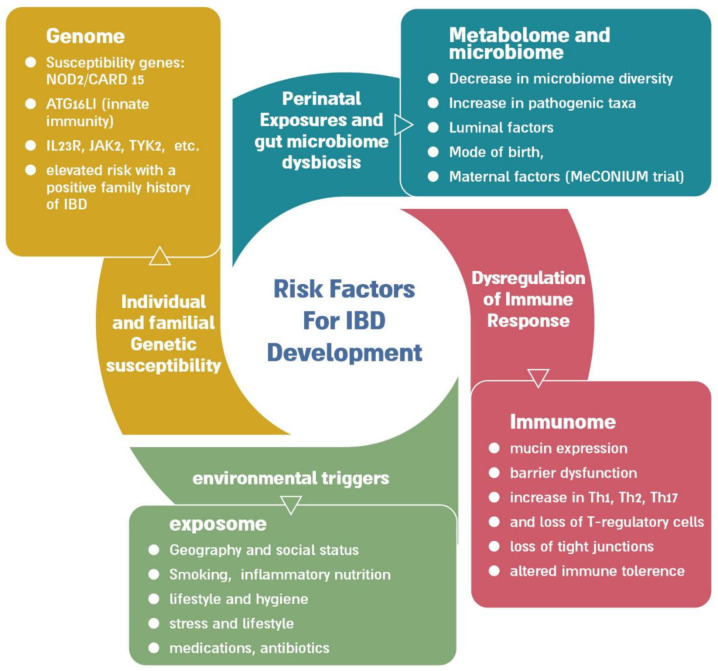
IBD is a multifactorial disease, arising from a complicated interplay between many factors, including genetics, epigenetics, disorders in the immune system, the microbiome, external environmental triggers, and more. All these variables can serve as future potential biomarkers, aiding in diagnosis and prognostication.

A schematic representation of the etiologies contributing to the development of IBD is presented below (Figure 1), all of which may serve as potential future diagnostic biomarkers:

## 5. The Implementation of IBD-Associated Biomarkers Can Be Considered across Various Stages of the Disease

### 5.1. Diagnostic Biomarkers

Due to consistent delays in the diagnosis of IBD, biomarkers that aid in the process of identifying patients at a high risk of developing IBD or those in the early stages of the disease are critical to promoting an ‘early head start’ in the treatment strategy in an attempt to prevent the development of complications and change the course of the disease [39]. While specific biomarkers for diagnosis have not yet been established, a variety of risk factors have been acknowledged (genetic mutations, positive family history, perinatal exposures, environmental exposures, lifestyle and hygiene, early life microbiome, and more) that increase the risk of developing IBD [14]. These risk factors can serve as potential targets for identifying diagnostic biomarkers and preventing the development of the disease in the future. Several large-scale projects studying the early life exposure effects on the development of IBD are in process, including the following: The MECONIUM study (Exploring MEChanisms Of disease traNsmission In Utero through the Microbiome) [40], the GEM Study (The Genetic, Environmental, Microbial) (Crohn’s and Colitis Canada, data not yet published) and more. These studies are expected to shed further light on the etiology and pathogenesis associated with the onset of IBD and help identify relevant diagnostic biomarkers.

### 5.2. Stratification and Prognostication Biomarkers

The IBD field has long sought to have the ability to precisely predict disease course at the time of diagnosis and respond accordingly by administering medications that are most effective for them as individuals (at the molecular level), assuming that this would maximize treatment response [41]. This strategy will enable patients to overcome the limitations of traditional medicine and will allow a shift in the emphasis from reactive to proactive management. In this approach, the medical decisions, practices, interventions, and/or products are tailored to the individual patient based on their predicted response or risk of disease [42]. In an attempt to differentiate the patients into subgroups (low vs. high risk for complications/bowel damage), it is necessary to identify validated biomarkers to aid in the stratification and prognostication process.

### 5.3. ‘Response to Treatment’ Biomarkers

The principal targets of IBD treatment, as outlined in earlier sections and in STRIDE guidelines, are to relieve symptoms, promote endoscopic mucosal healing, and prevent disease flare-ups; thus, predicting patients’ response to IBD therapy is crucial for avoiding long-term IBD-related complications, such as surgery and hospitalization. Additionally, the ability to predict patients’ response to treatment allows more individualized treatment options for patients, as many IBD patients become intolerant or stop responding to treatment over time [35]. The early identification of factors associated with clinical responses to therapies, such as immune markers, microbiome, anti-drug antibodies, and genetics, is crucial for treatment success when selecting or monitoring patients’ response to therapy.

## 6. Review of Current ‘Cutting-Edge’ Biomarkers in IBD, Consistent with the Origin of the Biomarker

### 6.1. Patient-Associated (Clinical) Biomarkers

Patient-associated factors (clinical biomarkers) can influence patients’ clinical trajectory and response to treatment. These factors can be anticipated in advance and, accordingly, be taken into consideration when contemplating therapeutic options. A classic example is smoking status. It has been well established that patients who are active smokers have a lower response to anti-TNFs and among responder’s shorter duration of response [43]. Additionally, rapid drug clearance and absorption issues are also influenced by patient characteristics (BMI, sex, CRP, albumin, prior immunogenicity) and should be considered when monitoring response to treatment [44].

#### Blood Based Biomarkers

Serological biomarkers can aid in patient stratification. The conventional serologic markers studied in the context of IBD are antibodies against bacterial antigens, such as ASCA and CBir1, antibodies against neutrophil antigens (pANCA), outer membrane porin C (Omp C), and I2 additional antibodies (novel homologs of the bacterial transcription-factor families) [45]. It has been demonstrated that an increase in serologic markers is associated with a more aggressive CD course [46]. Additionally, non-responders to anti-TNF and anti-integrin therapies show higher levels of IL-6, TNF-α, IL-1, IL-10, IL-8, and IFN-γ than responders [47].

Only one validated prognostic blood test44 has been developed to date, based on a gene-expression signature of CD8 + T cells in newly diagnosed, treatment-naive IBD patients. The currently ongoing PROFILE trial is evaluating this blood-based predictive biomarker in order to determine whether it can accurately influence the choice of the most suitable treatment regimen for each newly diagnosed CD patient. Patients are stratified according to their biomarker status in this trial [48].

Recently, the Olink multiplex panels were primarily used in a broader assessment of differentially expressed inflammatory proteins by serum proteome analysis (Olink Proteomics, Uppsala, Sweden). With the aid of these panels and a sizable cohort of prospectively included newly diagnosed IBD patients, the IBD Character biomarker research program identified a protein signature that significantly correlated with therapy escalation [49]. These findings will need to be verified by additional research, which will also illustrate the clinical significance of the found signature and serum proteome analysis in the prediction of clinical sub-phenotyping.

## 7. Serum Biomarkers Predicting Response to Treatment

### 7.1. Fecal Biomarkers

Encouragingly, in recent years, a novel key biomarker has been integrated into clinical practice and has transformed our everyday management of IBD patients. Fecal calprotectin (FC) is a 36 kDa calcium and zinc-binding protein that represents 60% of cytosolic proteins in granulocytes. The concentration of calprotectin in the feces is an indirect measure of neutrophil infiltration in the bowel mucosa, thus representing active inflammation [9,50]. Levels of FC are associated with IBD diagnosis, disease severity, response to treatment, disease relapse, and the need for colectomy in UC [51,52]. Studies have shown that FC has a pooled sensitivity and specificity for gut inflammation of 0.93 (0.85–0.97) and 0.96 (0.79–0.99), respectively, and a high negative predictive value (NPV) (0.96–0.98) [53]. FC has become so crucial in managing and monitoring IBD patients that it has recently been established as a formal IBD therapeutic goal in the recent STRIDE-2 study [54]. This successful evolution can serve as a motivational boost to further pursue the ‘biomarker search’ in the hope of finding additional novel biomarkers that can bring us closer to our goal of precision medicine.

However, it should be noted that FC is not a perfect biomarker and has some important drawbacks, including the following: the inability to differentiate between the distinct causes of inflammation in the bowel (including differentiating CD from UC), and an inaccurate correlation between the level of FC and the degree of inflammation in many patients [55].

In individuals with active UC, it has been discovered that other recently discovered fecal inflammatory indicators, such as the dimeric M2 isoform of pyruvate kinase (M2-PK), are reliable in predicting response to infliximab [55].

### 7.2. Tissue-Derived Biomarkers

The analysis of intestinal biopsies, which represent the site of active inflammation, has been an ongoing focus of biomarker discovery in IBD. The RISK study, where the authors revealed that an upregulated ileal extracellular matrix gene signature (derived from intestinal biopsies of newly diagnosed pediatric CD patients) is associated with the risk of developing a stricturing phenotype, brought to light the most significant prognostic findings from biopsy studies to date. The authors proposed a risk stratification model for complicated disease behavior based on clinical, serological, gene expression, and microbiological variables identified at CD diagnosis [56].

#### 7.2.1. Oncostatin M

Oncostatin M (OSM) has attracted much attention among possible tissue-derived targets and biomarkers. OSM is a member of the cytokine family interleukin (IL)-6, communicates through a receptor complex, and can activate a variety of signaling pathways. Inflamed intestinal tissue from IBD patients has been found to have extremely high levels of OSM and of the OSM receptor (OSMR) expression [57,58]. Due to the discovery that OSM is one of the most highly expressed cytokine genes in the mucosa of anti-TNF non-responders, OSM is regarded as a biomarker of illness diagnosis, of worse disease prognosis, and of non-responsiveness to anti-tumor necrosis factor (TNF) therapy [58]. Similarly, Intestinal α4β7 expression can theoretically predict therapeutic response to Vedolizumab (VDZ). It is hypothesized that intestinal α4β7 expression prior to VDZ therapy might represent an important biomarker that predicts therapeutic response to subsequent VDZ treatment [59].

#### 7.2.2. Expression of IL13RA2 (mRNA) in Mucosal Biopsies

Another breakthrough finding was demonstrated using microarray experiments. Researchers identified the expression of IL13RA2 (mRNA) in mucosal biopsies of IBD patients as a predictive marker for primary non responsiveness to infliximab (IFX) therapy, with a higher baseline expression in future non-responders [60]. This mucosal IL13RA2 predictive signal seems a robust marker, having been replicated in many independent cohorts. According to the findings, IL13RA2mRNA in intestinal mucosal tissues can be used as a biomarker to predict anti-TNF responsiveness and may even predict mucosal healing in IBD [61]. Furthermore, these results have been validated in patients treated with infliximab or adalimumab, but not with vedolizumab, thus reinforcing the specificity of these intriguing findings [61].

### 7.3. Genetic Biomarkers

#### 7.3.1. Genetic Profiling of Biomarkers

Genetic biomarkers have surfaced, in parallel with the advancement in the GWAS. Genetic factors are constant over time and are present before disease symptoms even develop, so they cannot be subjectively interpreted [62]. These traits make genetic markers valuable for an array of disease phases, including the following: patient stratification and prognostication, therapeutic decision making and the monitoring of responses to treatment, and even the patient’s level of risk in terms of responding to therapy.

In IBD patients, the genetic profiling of markers has demonstrated a positive association with the prediction of responses to biological therapy. The majority of genetic predictive markers are linked to cytokines or their receptors and immunoglobulin receptors, including genes for TNF/TNF-receptors, ATG16L1, apoptosis, NOD2/CARD15, CRP, IL23R and IL12, and genes associated with Fc receptors [63,64]. NOD-2 is a gene associated with complicated CD, and thus with the development of strictures and the need for surgery [65]. The “genome-wide polygenic risk score,” which incorporates the cumulative effects of genetic polymorphisms weighted by impact size in order to assess the probability of a specific trait depending on genotype, is another application of genetic biomarkers [66]. IBD was one of five diseases used to demonstrate this technique.

#### 7.3.2. HLA-DQA1*05 as a Genetic Predictor of Immunogenicity

The Personalizing Anti-TNF Therapy in the Crohn’s Disease (PANTS) study, which included 1240 biologic-naive CD patients from the UK, has revealed an intriguing finding that is highly likely to have clinical implications. It discovered that variations in the HLA-DQA1*05 allele are linked to a higher likelihood of developing antibodies against anti-TNF agents and that this immunogenicity can be significantly decreased by using a concurrent immunomodulator [67]. A future randomized controlled biomarker trial is necessary to determine whether pretreatment testing for HLA-DQA1*05 will improve patient outcomes by aiding physicians in selecting anti-TNF (with or without combination therapies), and whether it is feasible to precheck every patient.

#### 7.3.3. Prediction of Safety via Genetic Biomarkers

In terms of treatment safety, the likelihood of each individual patient experiencing potential side effects is a crucial element influencing therapy selection. One of the initial uses of genetics in IBD therapies was to identify patients who were more susceptible to developing side effects. The historical relationship between polymorphisms in the enzyme thiopurine methyltransferase [TPMT] and thiopurine-induced myelosuppression was successfully translated to mainstream clinical practice [68]. Many clinicians still use this tool in everyday practice before deciding on initiating therapy with thiopurines. GWAS analysis has also identified genetic factors that predispose individuals to amino salicylate-associated nephrotoxicity [69]. Genetic biomarkers can therefore be used in clinical practice to identify patients who are likely to experience adverse effects. Determining whether there are equivalent predictive markers for predicting intolerance or adverse effects to more novel biologic and/or small molecule agents will be the most important next step for personalized medicine in IBD.

#### 7.3.4. Prediction of Response to Treatment via Genetic Biomarkers

Several research projects have concentrated on the question of whether genetics might aid in the prediction of the patient’s responses to IBD therapy. In contrast to other fields such as oncology, where molecular markers have demonstrated substantial therapeutic value in predicting response rates to chemotherapy, genetic markers have unfortunately had limited success in predicting the outcomes of IBD therapy [62]. Advances in transcriptomic profiling (specifically combined with single-cell approaches) have been the focus of predictive biomarker development, especially at the tissue level. These advancements have enabled a much more granular insight into gene expression within inflammatory cells. For example, early microarray studies that examined the mucosal gene expression of patients with colonic CD or UC who were anti-TNF-naïve discovered four genes [IL13RA2, IL-11, IL-6, and TNFAIP6] that reliably distinguished infliximab responders from non-responders [70]. In a similar manner, the baseline expression levels of four other genes (PIWIL1, MAATS1, RGS13, and DCHS2) were found to predict the endoscopic response to vedolizumab [71]. The translation of complicated genetic markers into a straightforward test that can be utilized with ease in ordinary clinical treatment continues to be a significant barrier in applying these findings to everyday practice [41]. Therefore, while these findings are extremely intriguing, they are expensive, invasive, and far from being implemented into everyday clinical practice at this point.

### 7.4. The Microbiome as a Potential Biomarker

Due to the microbiome’s involvement in IBD pathogenesis, the taxonomic and functional composition of the gut microbiome may influence the likelihood of response to biological therapy. Recent advancements in metabolomic and metagenomic profiling have enabled further developments in discovering microbiome-associated biomarkers. It has been demonstrated that patients with a more diverse baseline microbiome show better responses to anti-TNF agents, vedolizumab, and ustekinumab [72]. The authors demonstrated significantly differentiated taxa between remission and non-remission groups. Another study showed that fewer mucus-colonizing bacteria, a higher abundance of short-chain fatty-acid-producing bacteria, and a lower abundance of pro-inflammatory bacteria are also associated with a favorable outcome [73]. These findings raise the hypothesis that early microbiome changes may be a marker of future patient responses to IBD treatment [72]. Moreover, models based on a combination of clinical data and microbiota have an excellent predictive power and hold great promise as future predictive tools.

### 7.5. Image-Based Biomarkers (Radiomics)

Findings at an initial scan, before the initiation of therapy, may prognosticate the disease course by recognizing underlying bowel damage due to long-term inflammation. One potential radiomic tool is the Lémann index [LI], which is a scoring system that uses clinical, endoscopic, and magnetic resonance enterography [MRE] information to assess cumulative disease burden. Bowel damage and the LI are independent prognostic factors for intestinal surgery (hazard ratio [HR]: 3.2) and of CD-related hospitalization [HR: 1.88] [74]. Similarly, small intestine oral contrast ultrasonography scores have been generated to evaluate numerous inflammatory intestinal wall characteristics that can be utilized as prognostic indices [75]. Within a 1-year follow-up, the CD sonographic lesion index may predict the need for surgery. Finally, there are constant attempts to utilize artificial intelligence to identify significant radiological markers with predictive value.

### 7.6. The Future: Omics-Inspired Biomarkers

The latest technological advances in healthcare and biology have paved the way for the systematic collection of complex data that can be used to support more classical and routinely collected information. Usually referred to as *multi-omics data*, these unprecedentedly large databases have the potential to provide integral information about DNA and RNA variations, protein abundance, gut microbiota, and many additional biological aspects to be interpreted within ‘mass’ clinical data [76]. This new level of ‘data abundance’ allows a more in-depth and granular analysis, increasing the chance of understanding complex traits. Consequently, the integration of profiles targeting different omics layers may provide another whole dimension in biomarker discovery.

#### Transcriptional Profiling

A few practical applications of multi-omics data are already in progress. Transcriptome analysis revealed an impairment of signaling cascades associated with the trafficking of white blood cells following VDZ therapy [59]. In non-responders to VDZ therapy, leukocyte-mediated inflammatory activity via the activation of TNF-dependent pathways was present, all of which were inhibited in responders to VDZ [59]. Furthermore, transcriptional risk scores integrating GWAS and expression quantitative trait locus data (the effects of genetic variation on gene expression) with ileal gene expression in a newly diagnosed CD cohort could be used to distinguish a complicated clinical course of CD [77]. The discovery of biomarkers may also be facilitated by peripheral blood transcriptomic profiling. Although conflicting findings have been published, baseline Triggering Receptor Expressed in Myeloid Cells-1 [TREM1] expression in both the gut and blood reliably indicated a poor response to anti-TNF medication [78,79]. These results demonstrate the potential of transcriptome analysis as a precision medicine tool.

**Figure 2:**
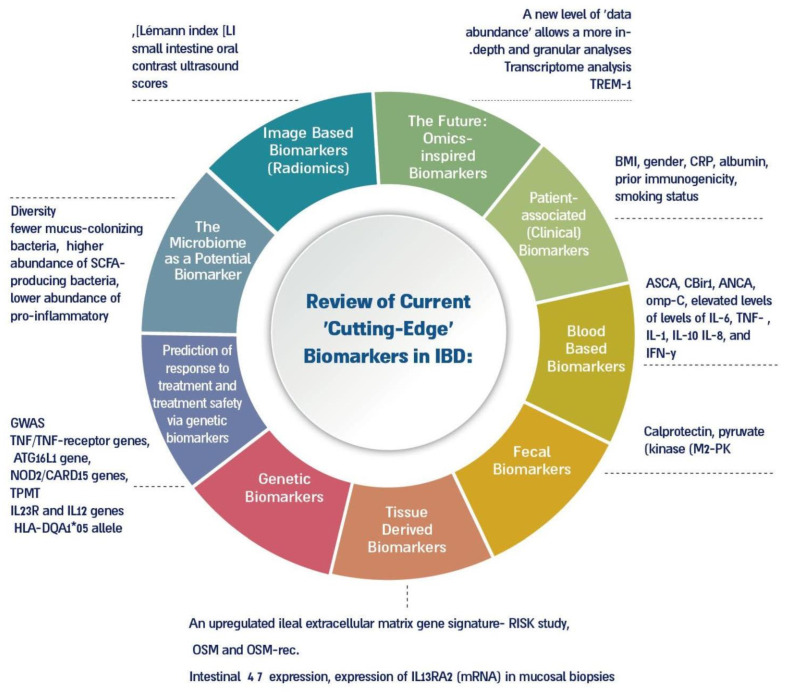
A schematic diagram depicting the current and future potential, multilayered, ‘state-of-the-art’ biomarkers utilized in IBD, categorized by origin and source of the biomarker.

A schematic representation of the current and future “cutting-Edge” novel biomarkers across the various etiologies they stem from is demonstrated in Figure 2.

## 8. Integrating Biomarkers in Everyday Clinical Practice—Reality or Wishful Thinking

The discovery and validation of a protein biomarker generally involve six stages: discovery, qualification, verification, assay optimization, clinical evaluation/validation, and commercialization [80]. In the past, a lack of prospective validation for promising biomarkers and models was a fundamental obstacle to the implementation of novel biomarkers. Interpreting data provided by mixtures of biomarkers and selecting from variations of therapies will likely present the greatest challenge for those precision medicine technologies that have been demonstrated to be validated and to have therapeutic relevance in a variety of groups [42]. 

In conclusion: exciting advancements in biomarker development have been made in the past years and positive preliminary validated results have been obtained implying that precision medicine that utilizes these biomarkers in clinical practice is hopefully nearby.

The present and future biomarkers and the leading studies incorporating them into clinical practice are summarized in Table 2. 

## 9. Putting the Pieces Together

### 9.1. Applying Precision Medicine in IBD Care—Uncovering the How, When, and Who

On a basic level, the personalization of medicine in IBD is already in existence. The use of clinical score models that incorporate patient characteristics in order to guide in choosing specific therapies and minimize complications has been employed in varying degrees for many years [76]. While these approaches are simple and do not constitute pure predictive medicine, they are important and should be incorporated into future modeling. In addition, there are currently used treatment strategies that aim for precision medicine and that have already been implemented in routine practice, including the following: defining individual (‘patient specific’) therapeutic targets in order to “treat to target” [34], applying therapeutic drug monitoring [85], and monitoring the patients with a policy of ‘tight control’, which warrants the close surveillance of symptoms, alongside inflammation biomarkers and more.

### 9.2. Summary: Precision Medicine in IBD: The Quest Continues

The growing understanding of the diverse molecular pathways that perpetuate inflammation in IBD, in combination with the ever-growing world of omics data, is expected to take us another step forward in our ability to integrate precision medicine into our everyday clinical practice. However, the gaps in our knowledge are still deep and there is still a long way to go in uncovering new molecular and microbiologic pathways and signals that participate in the inflammatory processes. Another crucial unmet need is in the novel biomarkers that are required to aid in diagnosing, stratifying, predicting the disease course, and monitoring treatments in IBD patients. However, impressive advancements have been made in the pursuit of new biomarkers at several distinct levels, including single-cell transcriptome analysis, microbiome and toxigenic profiling, whole tissue biopsies and genetic imprinting, novel serologic markers, radiomic (radiology profiling) and so much more. Thus, the years to come are expected to shed light on the pathogenesis of IBD and help us detect useful biomarkers that can be employed to our advantage.

Our therapeutic success in IBD is suboptimal. This is due, in part, to the heterogeneity and complexity of the disease, so specific therapies may not be suitable for specific patients. The development of therapies based on an individual’s underlying genetic, epigenetic, or microbial profile would help deliver the goal of personalized medicine in IBD, with a greater selection of medications to match the underlying problem leading to inflammation in an individual. Hopefully, the future will hold great promise for IBD patients in the form of treatments tailored to individual patients and not to molecular pathways.

Yet, beyond the ever-evolving world of biomarkers, genetics, and vast omics-data, we must reinforce our basic clinical everyday methods in the form of tight control, ongoing patient assessment, addressing patients’ mental health status, bestowing nutritional support, and much more. Only by combining multifold layers of treatment, from the granular molecular level to everyday clinical practice, can we proceed to make the vision of precision medicine a reality.

## Figures and Tables

**Table 1:** The unique and diverse characteristics of the gut microbiota in IBD patients.

**Dysbiosis**	Reduced microbial diversity and increased abundance of certain bacterial species	[30]
**Firmicutes/Bacteroidetes ratio**	Increased Firmicutes/Bacteroidetes ratio in IBD patients	[31]
**Bacterial species**	Increased abundance of bacterial species, including Escherichiacoli, Enterobacteriaceae, and Fusobacterium, in IBD patients	[31]
**Viruses**	Increased abundance of viruses, including bacteriophages, in IBD patients	[32]
**Fungi**	Increased abundance of fungi, including Candida, in IBD patients	[32]
**Metabolites**	Altered production of metabolites, such as short-chain fatty acids and mucin, in IBD patients	[31]

**Table 2:** The present and future potential biomarkers integrated into IBD practice, categorized by the specific intent of the biomarkers.

Biomarkers	Description	Exemplars	Leading Studies	References
Diagnostic biomarkers	Biomarkers aid in the process of identifying patients at high risk to develop IBD or in early diagnosis of ibd	Genetic mutations, Perinatal exposures, Environmental Exposures Early life microbiome	Meconium Study Gem Project	[10,45,80]
Stratification and Prognostication Biomarkers	Separates patients into discrete groups early in the course of the disease in an attempt to support treatment decisions.	Oncostatin M (OSM) and OSM receptor NOD-2 OMP-C ASCA\ANCA	The Genome-wide polygenic risk score	[44,49,51,59,60]
		A prognostic blood test derived from a gene-expression signature of CD8 + T cells	PROFILE trial	
		An ECM-derived signature derived from intestinal biopsies of newly diagnosed pediatric CD patients	The RISK study	
‘Predicting response to treatment’ biomarkers	Biomarkers predicting response to treatment, preferably specific to the mode of action, aiding in treatment decisions	OSM and OSM receptors serum concentrations of IL22 Smoking status BMI TREM-I		[60,62,73,74,81]
Treatment safety biomarkers	Biomarkers aimed to identify patients at higher risk to develop side effects	TPMT HLA-DQA1*05	PANTS study	[48,70]
Treatment response biomarkers	Noninvasive biomarkers aid in evaluating treatment response, thus reducing the need for invasive assessments	Serum CRP Fecal calprotectin Therapeutic drug monitoring Multi-omics data	CALM study	[37,53,75,82,83,84]
